# Rhinovirus-induced anti-viral interferon secretion is not deficient and not delayed in sinonasal epithelial cells of patients with chronic rhinosinusitis with nasal polyp

**DOI:** 10.3389/fimmu.2022.1025796

**Published:** 2022-10-21

**Authors:** Sang Hag Lee, Mun Soo Han, Tae Hoon Lee, Da Bin Lee, Jae Hyung Park, Seung Hyeok Lee, Tae Hoon Kim

**Affiliations:** Department of Otorhinolaryngology-Head & Neck Surgery, College of Medicine, Korea University, Seoul, South Korea

**Keywords:** rhinovirus, interferon, replication, chronic rhinosinusitis, epithelial cells, TLR3, RIG-1, MDA5

## Abstract

Dysregulated innate and adaptive immune response to rhinoviral infection plays an important role in the exacerbation or progressive course of chronic rhinosinusitis (CRS). However, few studies have evaluated whether rhinovirus-induced production of anti-viral interferon is deficient or delayed in inflammatory epithelial cells of patients with CRS with nasal polyps. The aim of the present study is to investigate the replication rates of rhinovirus 16 (RV 16), RV16-induced antiviral interferon secretion, and the expression levels of pattern recognition receptors after RV 16 infection or TLR3 stimulation with poly (I: C) in normal and inflammatory epithelial cells. Inflammatory epithelial cells were obtained from CRS patients with nasal polyps and normal epithelial cells were derived from ethmoid sinus mucosa during endoscopic reduction of blowout fracture or uncinate process mucosa of patients with septal deviation. Cultured cells were infected with RV 16 or treated with poly (I: C) for 24, 48, and 72 h. Cells and media were harvested at each time point and used to evaluate RV16 replication rates, the secretion of IFN-β, -λ1, -λ2, viperin, Mx, and OAS, and the expression levels of TRL3, RIG-I, MDA5, phospho-NFκB, and phospho-IRF3. RV replication rates reached peak levels 48 h after inoculation in both normal and inflammatory epithelial cells and showed no difference between both groups of epithelial cells at any time point. The release of IFN-β, -λ1, and -λ2 in normal and inflammatory epithelial cells was also strongly induced 48 h after RV16 inoculation but reached peak levels 24 h after poly (I: C) treatment. The expression levels of viperin, Mx, OAS, TLR3, RIG-I, MDA5, phospho-NFκB, and phospho-IRF3 showed similar patterns in both groups of epithelial cells. These results suggest that the production of RV16-induced antiviral interferons is not deficient or delayed in inflammatory epithelial cells from CRS patients with nasal polyps.

## Introduction

Chronic rhinosinusitis (CRS) is an inflammatory disorder associated with persistent and heterogenous inflammation of sinus mucosa lining the paranasal sinuses and is frequently exacerbated (1). Clinically, respiratory viral infections are implicated as a trigger for CRS development, enhancing the disrupted intercellular junction of the sinonasal epithelium associated with increased epithelial permeability and contributing to bacterial mucosal adhesion (2–5). Thus, it has been suggested that dysregulated immune responses to the invasion of microorganisms, including viruses, underlie the persistence of the inflammatory state (2–6). Nevertheless, the role of impaired anti-viral interferon response in respiratory viral infections needs to be elucidated in the development and exacerbation of CRS.

During the early phases of respiratory viral infection, viral nucleic acids in epithelial cells are detected and engaged by three pattern-recognition receptors including Toll-like receptor (TLR)-3, retinoic acid-inducible gene (RIG)-I, and melanoma differentiation-associated protein (MDA)-5 (7–9). Activation of these receptors enhances the release of anti-viral interferons to combat viral infection, activating NF-κB and interferon regulatory factor 3 (IRF3) (10–12).

In contrast to the upper respiratory tract, respiratory viral infections in bronchial epithelial cells have been studied extensively, and data have shown that a reduced anti-viral immune response leads to increased susceptibility to rhinovirus (RV) infections in asthmatics or chronic obstructive pulmonary disease (COPD) patients (13–17). However, a recent study reported that the innate anti-viral response of epithelial cells against RV was consistent across healthy subjects, asthmatics, and COPD patients, but was delayed in asthmatics or patients with COPD compared with healthy subjects (16). We previously reported decreased expression levels of IFN-β, -λ1, and -λ2, and IFN-stimulated genes (ISGs) including viperin, Myxovirus resistance (Mx), and oligoadenylate synthase (OAS) in CRS, suggesting that they may contribute to deficient antiviral innate responses in CRS (18). However, whether RV-induced production of anti-viral interferons is deficient or delayed in chronic rhinosinusitis with nasal polyps (CRSwNP) has not been determined.

In the present study, we aim to (1) evaluate whether there are differences in rhinovirus 16 (RV 16) replication rates between healthy subjects and patients with CRSwNP; (2) determine whether the production of anti-viral interferons in cells infected with RV 16 is deficient in patients with CRSwNP; and (3) investigate whether the expression of pattern recognition receptors, phosphorylated NF-κB and IRF3, key signaling molecules implicated in RV-induced anti-viral interferons, is deficient in patients with CRSwNP after RV 16 infection and poly (I:C) treatment.

## Materials and methods

### Subjects

Non-inflammatory sinonasal mucosal tissues were collected from the uncinate processes of patients with septal deviation (n = 50) during surgery and from the ethmoid sinus cavity during endoscopic intranasal reduction of blowout fracture (n = 15) and were considered healthy mucosa or controls. Inflammatory mucosal tissues were obtained from the ethmoid sinus mucosa of patients with CRSwNP during endoscopic sinus surgery (n = 59) (**Supplementary Table 1**).

All participants provided signed informed consent. This study was carried out under the ethicals guideline of our institution. Patients with allergic rhinitis, aspirin hypersensitivity, asthma, or a history of sinus surgery were excluded. None of the subjects were treated with any medication, including antibiotics, steroids, or antihistamines for at least three months before surgery.

### Isolation of sinonasal epithelial cells and air-liquid interface culture

Healthy and inflammatory sinonasal epithelial cells were isolated by enzymatic degradation of the sinonasal mucosa obtained from controls and patients with CRSwNP and were then cultivated in 6 well plates (SPL, Pocheon City, Korea) supplemented with bronchial epithelial cell medium (BEpiCM; ScienCell Lab, CA, USA). Once the cells in submerged cultures were confluent, second-passage cells were seeded at 1 × 10^5^ cells per insert and grown under an air-liquid interface (ALI) until the epithelial cells reached full confluence. ALI culture was maintained for 4 - 6 weeks and then used for the experiments.

### Propagation of RV 16

Human RV 16 (ATCC VR-283PQ, VA, USA) was propagated in H1HeLa cells (ATCC) cultured in flasks supplemented with minimum essential medium (MEM, Thermo Fisher Scientific) at 33°C according to ATCC recommendations. Thereafter, a cytopathic effect was observed by measuring 50% tissue culture infective doses (TCID_50_).

### RV 16 infection, poly (I: C) treatment, and sampling

ALI cultures were infected apically with RV 16. Cultured cells were washed with DPBS to clear mucus secreted from epithelial cells prior to RV 16 infection. RV 16 stock, diluted to obtain a multiplicity of infection (MOI) of 0.5, was inoculated to the apical surface of ALI cultures and incubated for 5 h in 150 μL MEM at 33°C, 5% CO_2_. Thereafter, the apical surface was rinsed thrice with PBS to remove any non-attached virus. After the final wash, the apical and basal spaces were replaced with fresh ALI medium and were incubated for different time points at 33°C. We previously confirmed that an MOI of 0.5 does not enhance cell death, and this MOI was selected as a viral inoculation dose.

To analyze the expression levels of mRNAs and proteins as well as the replication rate of RV 16 during the experimental period, basal media and cells harvested at 24, 48, and 72 h after inoculation were kept in a deep freezer at -75°C.

Viral RNA was extracted from cultured cells at each time point using a QIAamp Viral RNA Mini Kit (Qiagen, Ontario, Canada). HRV 16 Genesig standard kit^®^ (Primerdesign™ LTD., Chandler’s Ford, UK) was used for the analysis of RV 16 RNA levels by RT-qPCR. Standard curves for the quantification of RV 16 were made by RV16 RNA standards included in the kit.

In addition, ALI cultures were also incubated with a TLR3 agonist (poly (I: C), 10 μM, *In vivo*Gen, CA, USA) for 24, 48, and 72 h. Thereafter, basal media and cells obtained at each time point were kept at -75°C for mRNA or protein analysis.

The expression levels of IFN-β, -λ1, and -λ2 mRNA transcripts and proteins were measured in cultured cells and basal media harvested at each time point by RT-qPCR and ELISA. RT-qPCR and western blotting were carried out to evaluate the expression levels of Viperin, Mx, OAS, TLR3, RIG-I, and MDA5 mRNA transcripts and proteins in cells obtained at each time point. NF-κB, phospho-NF-κB, IRF3, and phospho-IRF3 levels were evaluated by western blotting. Cytotoxicity was determined with a lactate dehydrogenase (LDH) assay kit (Abcam, Cambridge, United Kingdom).

### RT-qPCR and western blot

Total RNA was extracted from the cells harvested at each time point with Qiazol lysis reagent (QIAZEN Inc, CA, USA) and then reverse transcribed into cDNA using a reaction fluid mixed with sense and anti-sense primer, oligo dT primer (GenDEPOT, TX, USA), and MML-V (Invitrogen). Subsequently, RT-qPCR based on SYBR-Green (Takara bio, Shiga, Japan) was carried out using cDNA. A NCBI – Primer Blast software was used to find the primers of each genes. The primer sequences of genes were described in **Table 1**.

**Table 1:** Primers used for real time PCR analysis.

Primer	Sequence	
IFN-β	S:5′- GCACAACAGGTAGTAGGCGA-3′	AS: 5′- TGGAAAGAGCTGTCGTGGAG -3′
IFN-λ1	S:5′- GGTGACTTTGGTGCTAGGCT-3′	AS: 5′- GGCCTTCTTGAAGCTCGCTA -3
IFN-λ2	S:5′- GTGACAGCCTCAGAGTGTTTCT-3′	AS: 5′- AACTGCTCCAGTCACGGTCA -3′
Mx	S:5′- CAGCTCAGGGGCTTTGGAAT-3′	AS: 5′- CCTTGGAATGGTGGCTGGAT -3′
OAS	S:5′- GCTGAGGCCTGGCTGAATTA -3′	AS: 5′- CAGTCCTCTTCTGCCTGTGG -3′
TLR 3	S:5′- AGTGCCGTCTATTTGCCACA -3′	AS: 5′- GCATCCCAAAGGGCAAAAGG -3′
RIG-1	S:5′- AGAGCACTTGTGGACGCTTT -3′	AS: 5′- TGTTTTGCCACGTCCAGTCA -3′
MDA5	S: 5′- TTGGACTCGGGAATTCGTGG -3′	AS: 5′- AGCTCAGGGTTCATGTAGCG -3′
GAPDH	S:5′- CCACATCGCTCAGACACCAT -3′	AS: 5′- AGTTAAGAACAGCCCTGGTGA -3′

For western blotting, the cultured cell was lysed in RIPA buffer (GenDEPOT). 25 μg of denatured proteins were electrophoresed using SDS-PAGE and then, were transferred onto PVDF membranes (BioRad, MA, USA) and followed by blocking of the membrane with 5% skim milk. Thereafter, the membranes were incubated overnight at 4°C with the primary antibodies against β actin (catalog no. sc-8432, Santa Cruz, USA), viperin (catalog no. #13996, Cell Signaling, USA), OAS1 (catalog no. #14498, Cell Signaling, USA), Mx1 (catalog no. #378498, Cell Signaling, USA), TLR3 (catalog no. #6961, Cell Signaling, USA), RIG-I (catalog no. #4200, Cell Signaling, USA), MDA5 (catalog no. #5321, Cell Signaling, USA) NF-κB (catalog no. #8242, Cell Signaling, USA), phospho-NF-κB (catalog no. #3033, Cell Signaling, USA), IRF3 (catalog no. #11904, Cell Signaling, USA), phospho-IRF3 (catalog no. #37829, Cell Signaling, USA). Specific protein bands were visualized by an enhanced chemiluminescence machine (iBright CL1000, ThermoFisher scientific, USA) and quantified with Image J software (Ver. 1.51).

### Determination of IFN-β, -λ1, and -λ2 by ELISA

Basal media were used for the analysis of IFN-β, -λ1, and -λ2 by ELISA (R&D Systems).

### Statistics

Statistical analysis was conducted with the SPSS package (ver 16.0.0 for Windows; SPSS Inc, Chicago, IL, USA). One-way analysis of variance was performed to compare RT-qPCR, ELISA, and western blot data. Kruskal-Wallis test with Dunn’s *post-hoc* test was employed for multiple comparisons to compare differences between time points. Data were represented as a mean ± standard error. P values < 0.05 were regarded statistically significant.

## Results

**Figure 1:**
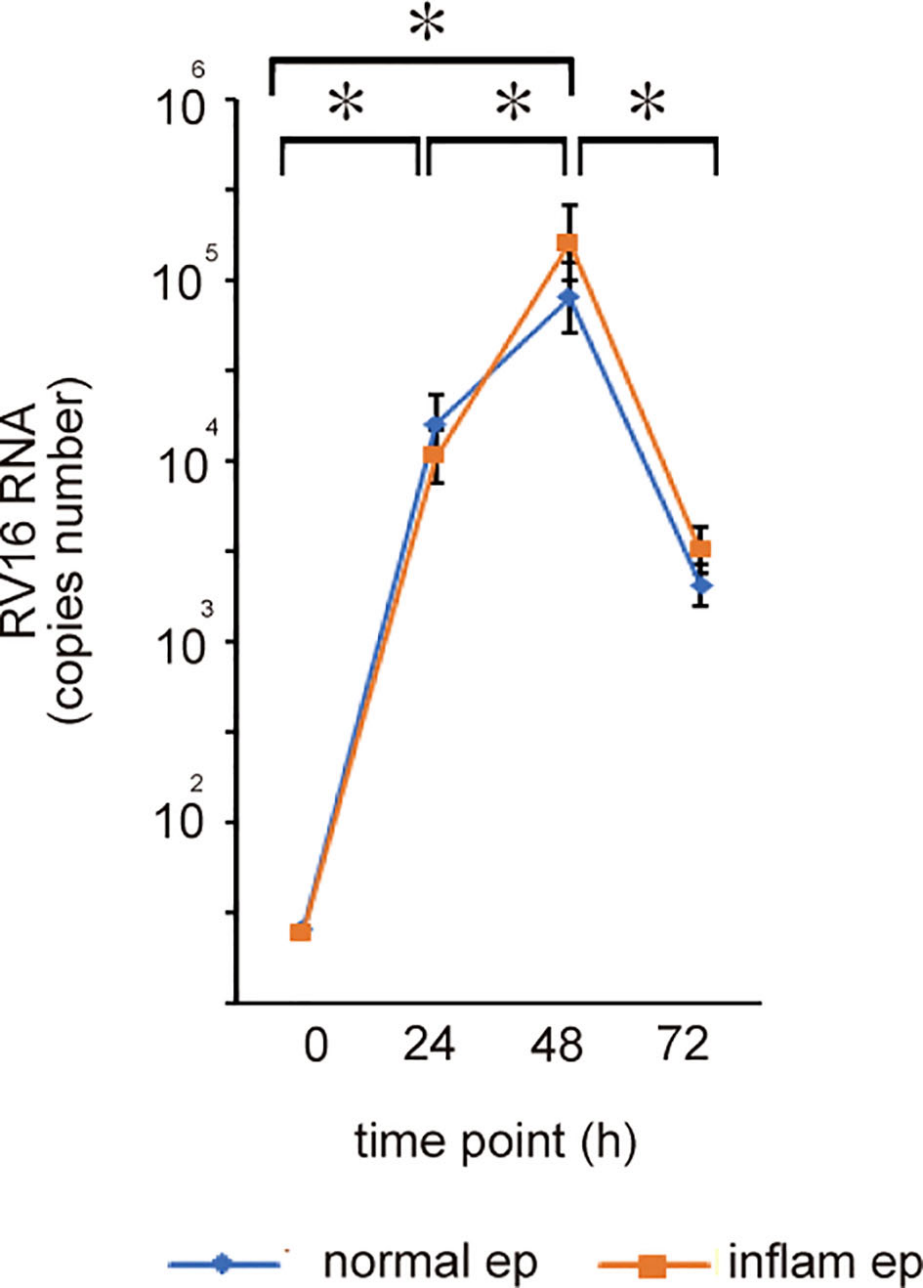
Normal and inflammatory sinonasal epithelial cells obtained from healthy control (N=7) and patients with CRSwNP (n=7) respectively were cultured in ALI which was infected with rhinovirus (RV)16 at MOI of 0.5. RV replication rates were assessed by RT-qPCR 24, 48, and 72 h after RV 16 inoculation and expressed as total copy number. The copy number of RV 16; normal epithelium; 24 h: 1.1 x 10 ^4^ – 5 x 10 ^4^, 48 h: 8.5 x 10^4^ – 1.1 x 10 ^5^, 72 h: 3.4 x 10^3^ – 4.2 x 10 ^3^, inflammatory epithelium: 24 hr: 0.92 x 10^4^ – 3.3 x 10^4^, 48 hr: 9.5 x 10 ^4^ – 3.2 x 10 ^5^, 72 hr: 3.7 x 10 ^3^ – 4.6 x 10 ^3^). *indicates a significant difference between each time-points.

To assess whether RV16 exposure at an MOI of 0.5 could infect cultured epithelial cells, intracellular RV 16 RNA levels were monitored over time by RT-qPCR and expressed as total copy numbers. The levels of RV 16 replication were detected 24-72 h after inoculation in normal epithelial cells and a peak viral titer was observed 48 h post-infection (**Figure 1**). These findings were also observed in cultured inflammatory epithelial cells derived from patients with CRSwNP (**Figure 1**). Furthermore, the total copy number of RV 16 assessed at each time-point did not differ between both groups of epithelial cells; normal vs inflammatory epithelial cells (**Figure 1** and **Supplementary Figure 1**). Infection with RV 16 did not affect cell viability as determined by LDH assay in both groups of cells (data not shown). These data indicate that RV 16 proliferation is not significantly higher in inflammatory sinonasal epithelial cells than in normal epithelial cells.

**Figure 2:**
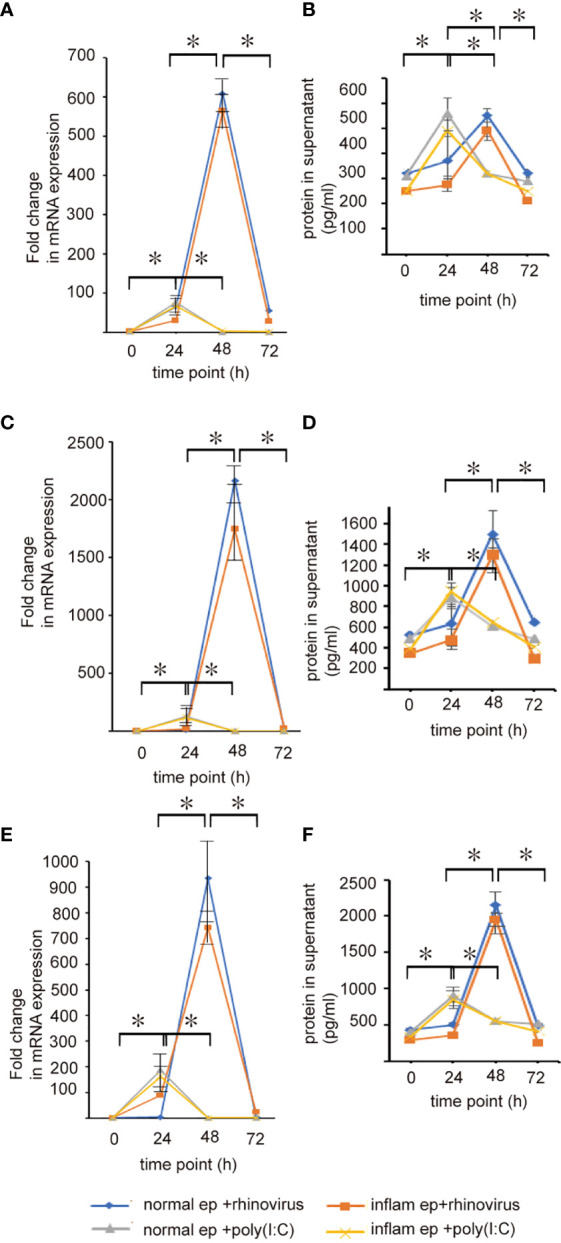
The expression levels of IFN-β **(A, B)**, IFN-λ1 **(C, D)**, and IFN-λ2 **(E, F)** mRNAs and proteins in basal media and cultured sinonasal epithelial cells 24, 48, and 72 h after RV 16 inoculation and poly(I: C) treatment, which were evaluated by RT– qPCR **(A, C, E)** and ELISA **(B, D, F)**. Data are mean ± SEM from 7 different epithelial donors. Normal ep + rhinovirus indicates that cultured normal epithelial cells were infected with RV. Inflam ep + rhinovirus indicates that cultured inflammatory epithelial cells were infected with RV. Normal ep + poly (I: C) means poly (I: C)-treated cultured normal epithelial cells. Inflam + poly (I:C) means poly (I: C)-treated cultured inflammatory epithelial cells. * indicates a significant difference between each time-points.

**Figure 3:**
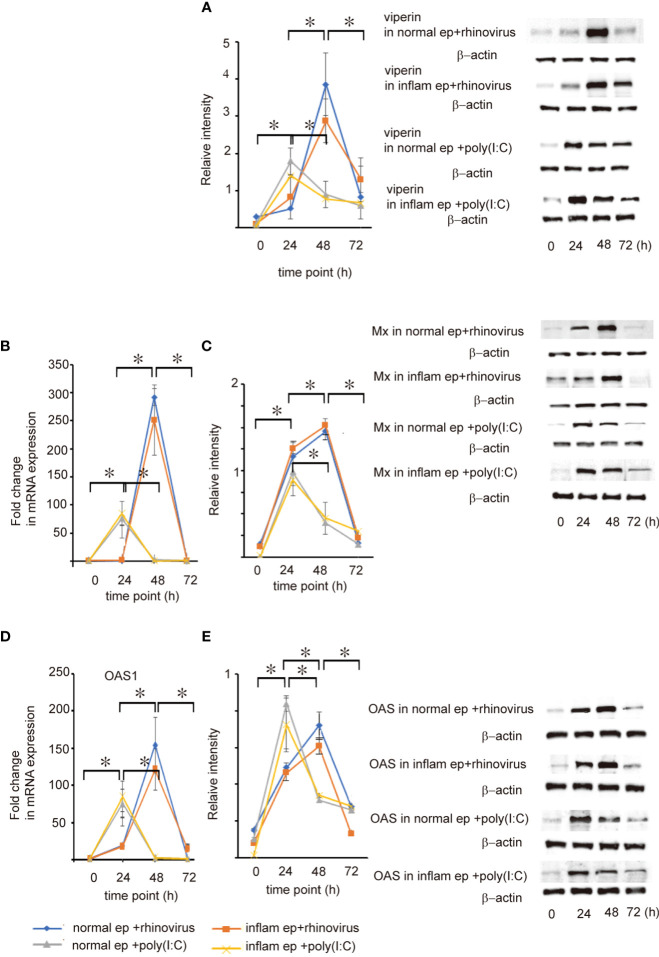
The expression level of viperin, Mx, and OAS mRNA and protein expression in cultured normal and inflammatory sinonasal epithelial cells obtained from healthy control and patients with CRSwNP, respectively. Their levels were evaluated 24, 48, and 72 h after RV 16 inoculation and poly (I: C) treatment, which were evaluated by RT– qPCR **(B, D)** and western blot **(A, C, E)**. Right panels located in the figure show representative protein bands evaluated with western blot. Data are mean ± SEM from 7 different epithelial donors. Normal ep + rhinovirus indicates that cultured epithelial cells were infected with RV. Inflam ep + rhinovirus indicates that cultured inflammatory epithelial cells were infected with RV. Normal ep + poly (I: C) means poly (I: C)-treated cultured normal epithelial cells. Inflam + poly (I:C) means poly (I:C)-treated cultured inflammatory epithelial cells. * indicates a significant difference between each time-points.

To evaluate whether anti-viral interferon production in sinonasal mucosa is deficient or delayed in patients with CRSwNP, the production of anti-viral interferon was measured in normal and inflammatory sinonasal epithelial cells infected with RV 16 or treated with poly (I: C) for 72 h. RV 16 infection at an MOI of 0.5 also enhanced the release of IFN-β, -λ1, and -λ2, and ISGs in both groups of epithelial cells (**Figures 2**, **3** and **Supplementary Tables 2**, **3** and **Supplementary Figures 2**-**5**). The mean concentrations of IFN-β, -λ1, and -λ2 in both groups of epithelial cells were detectable at 24 - 72 h after RV 16 inoculation, with peak induction observed at 48 h post-infection, showing patterns similar to RV 16 replication rates (**Figures 2B, D, F** and **Supplementary Tables 2**, **3** and **Supplementary Figures 3**). In parallel with protein secretion, IFN-β, -λ1, and -λ2 mRNA expression levels were strongly induced by RV 16 infection at 48 h in both group of epithelial cells (**Figures 2A, C, E** and **Supplementary Tables 2**, **3** and **Supplementary Figures 2**). However, the expression levels of IFN-β, -λ1, and -λ2 mRNA and protein that was measured at each time -point did not significantly differ between both groups of epithelial cells (**Figure 2** and **Supplementary Tables 2**, **3** and **Supplementary Figures 2**, **3**).

In poly (I: C)-treated normal epithelial cells, the expression levels of IFN-β, -λ1, and -λ2 mRNA transcripts and proteins were detected 24 - 48h after treatment. In contrast to RV 16 infection, their expression reached a peak at 24 h post-treatment (**Figures 2A–F** and **Supplementary Figures 2**, **3**). These findings were also noted in poly (I: C)-treated inflammatory epithelial cells. (**Figures 2A–F** and **Supplementary Figures 2**, **3**). Furthermore, their expression levels evaluated at 24 h post-treatment did not differ between normal and inflammatory epithelial cells (**Figures 2A–F** and **Supplementary Figures 2**, **3**). Taken together, these results indicate that anti-viral interferon secretion is not delayed or not deficient in inflammatory epithelial cells infected with RV 16 and stimulated with TLR 3 agonist. (**Figure 2** and **Supplementary Figures 2**, **3**).

Secretion of viperin, Mx, and OAS in both groups of epithelial cells was induced after RV 16 infection or poly(I: C) treatment (**Figure 3** and **Supplementary Figures 4**, **5**). In parallel with IFN-β, -λ1, and -λ2 release, the expression of viperin, Mx, and OAS was strongly induced by RV 16 at 48 h after RV16 infection in both groups of epithelial cells whereas their expression reached the peak at 24 h after treatment with poly(I: C) (**Figure 3** and **Supplementary Figures 4**, **5**). These results indicate that the secretion of ISGs after RV 16 infection or poly (I: C) treatment was not delayed in inflammatory epithelial cells compared with normal epithelial cells. Furthermore, their expression levels did not differ at any time point between normal and inflammatory epithelial cells, suggesting that anti-viral induction of ISGs is not deficient in patients with CRSwNP.

**Figure 4:**
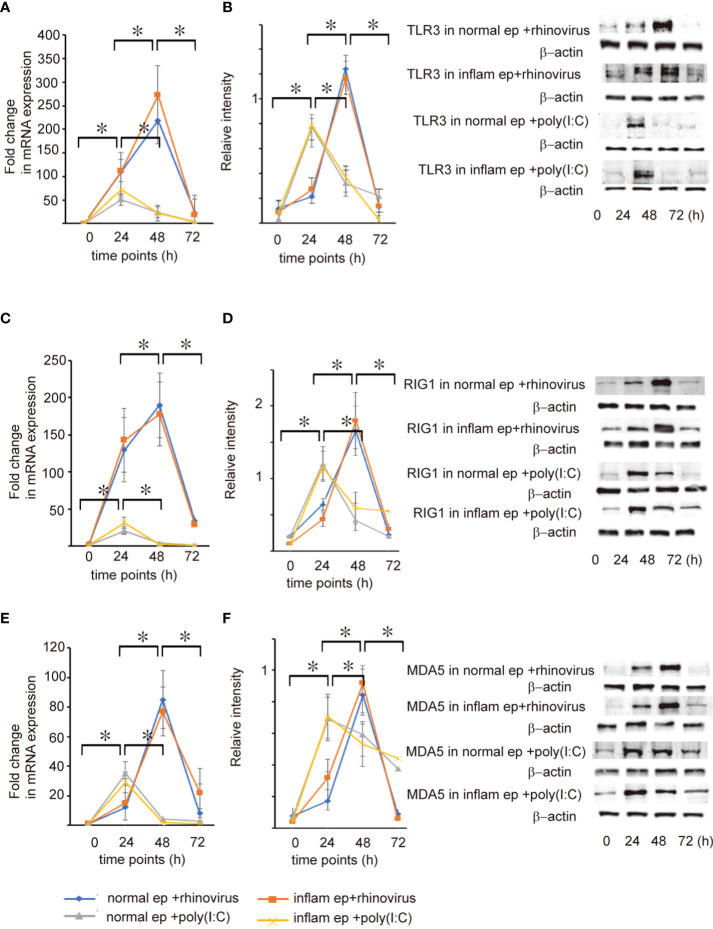
The expression level of TLR3 **(A, B)**, RIG-I **(C, D)**, and MDA5 mRNA **(E, F)** and protein expression in cultured normal and inflammatory sinonasal epithelial cells obtained from healthy control and patients with CRSwNP, respectively. Their levels were evaluated 24, 48, and 72 h after RV 16 inoculation and poly(I: C) treatment, which were evaluated by RT – qPCR **(A, C, E)** and western blot **(B, D, F)**. Right panels located in the figure show representative protein bands evaluated with western blot. Data are mean ± SEM from 7 different epithelial donors. Normal ep + rhinovirus indicates that cultured normal epithelial cells were infected with RV 16. Inflam ep + rhinovirus indicates that cultured inflammatory epithelial cells were infected with RV 16. Normal ep + poly (I: C) means poly(I: C)-treated cultured normal epithelial cells. Inflam + poly (I:C) means poly (I:C)-treated cultured inflammatory epithelial cells. * indicates a significant difference between each time-points.

**Figure 5:**
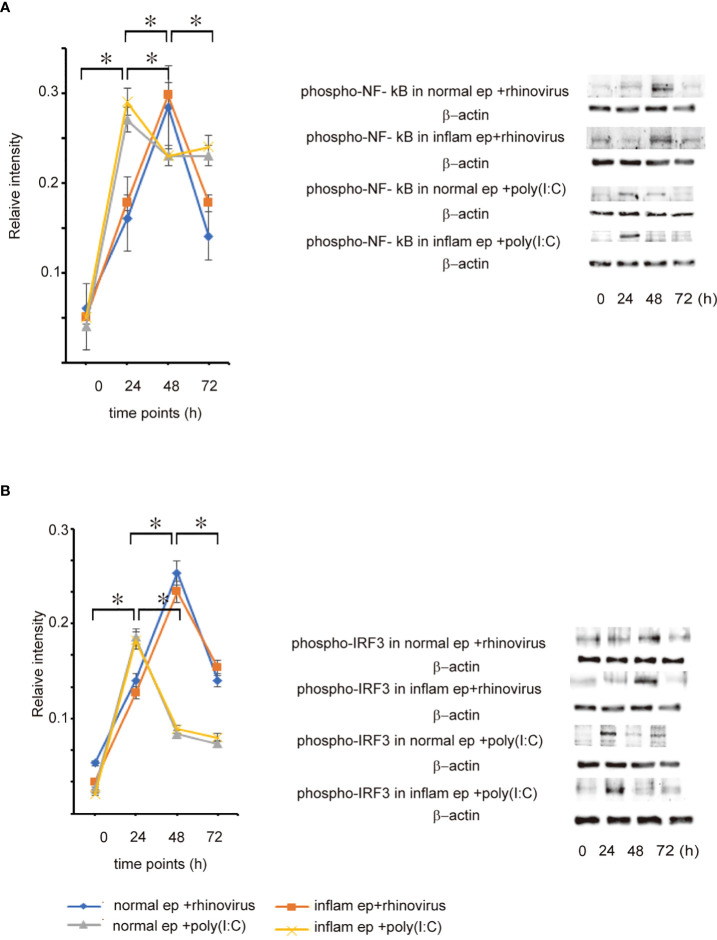
The expression level of phospho-NF-κB **(A)** and phosphor-IRF3 **(B)** protein in cultured normal and inflammatory sinonasal epithelial cells obtained from healthy control and patients with CRSwNP, respectively. Their levels were evaluated 24, 48, and 72 h after RV 16 inoculation and poly (I: C) treatment, which were evaluated by western blot. Right panels located in the figure show representative protein bands evaluated with western blot. Data are mean ± SEM from 7 different epithelial donors. Normal ep + rhinovirus indicates that cultured normal epithelial cells were infected with RV 16. Inflam ep + rhinovirus indicates that cultured inflammatory epithelial cells were infected with RV 16. Normal ep + poly (I: C) means poly(I: C)-treated cultured normal epithelial cells. Inflam + poly (I:C) means poly (I:C)-treated cultured inflammatory epithelial cells. * indicates a significant difference between each time-points.

Pattern recognition receptors recognize viral components produced during RV replication and subsequently activate NF-κB and IRF3, leading to the secretion of anti-viral interferon (10–12). To evaluate whether the expression levels of pattern recognition receptors were deficient or delayed in patients with CRSwNP after RV infection, the expression levels of TRL3, RIG-I, and MDA5 were estimated during the time-course by RT-qPCR and western blot assays. When infected with RV 16, the expression levels of TLR3, RIG-I, and MDA5 mRNA and protein tended to increase over time in both groups of epithelial cells and reached peak levels at 48 h post-infection, showing a similar pattern to that of RV16 replication (**Figure 4** and **Supplementary Figures 6**, **7**). Poly (I: C) treatment induced significant expression of TLR3, RIG-I, and MDA5 mRNA and protein at 24 h, compared with 12 and 48 h in both groups of epithelial cells (**Figure 4** and **Supplementary Figures 6**, **7**); however, their levels did not differ between both type of epithelial cells at any time point (**Figure 4** and **Supplementary Figures 6**, **7**). Furthermore, we confirmed that up-regulation of phospho-NF-κβ and phospho-IRF3 in both groups of epithelial cells was strongly observed 48 h after RV16 infection and 24 h after poly(I: C) treatment (**Figure 5**, **Supplementary Figure 8**). As expected, the expression levels of phospho-NF-κβ and phospho-IRF3 did not significantly differ between both groups of epithelial cells at any time point (**Figure 5**, **Supplementary Figure 8**).

## Discussion

An impaired anti-viral immune response through the interferon response has been proposed as a mechanism for susceptibility to RV infection in patients with respiratory disorders such as asthma, COPD, and cystic fibrosis (13–17, 19–22). We previously reported the decreased expression levels of IFN-β, -λ1, -λ2, and ISGs in inflammatory sinonasal mucosa of patients with CRSwNP compared with those of normal mucosa (18). Therefore, to determine if the anti-viral interferon pathway participates in the RV-associated exacerbation and development of CRS, we performed a detailed kinetic analysis for RV 16 replication rates, secretion of anti-viral interferons, and expression levels of pattern recognition receptors in both cultured normal and inflammatory epithelial cells after RV 16 infection and poly (I: C) treatment. The results suggest that the anti-viral interferon response to RV 16 infection is not delayed and is not deficient in patients with CRSwNP compared with healthy controls.

To explore whether the decreased expression of IFN-β, -λ1, -λ2, and ISGs in patients with CRSwNP can enhance the replication of RV 16 in inflammatory epithelial cells of patients with CRSwNP, we evaluated RV 16 replication rates in normal and inflammatory sinonasal epithelial cells from normal control and patients with CRSwNP. RV 16 replication rates in both groups of epithelial cells (normal and inflammatory epithelial cells) rapidly increased 24 h after RV 16 inoculation, reaching peak levels at 48 h post-inoculation. Furthermore, the total copy numbers did not differ between both groups of epithelial cells at any time point. Therefore, these results indicate that RV 16 replication rates are not higher in patients with CRSwNP compared with healthy subjects, suggesting that viral infection or replication in sinonasal epithelial cells was not significantly altered by inflammatory status, at least in CRSwNP. Similarly, previous studies on RV infection documented that peak viral RNA levels were detected in bronchial epithelial cells 24 -48 h after RV-A1 infection at multiple doses, ranging from 0.001 to 1. After RV-A1 infection at an MOI of 0.001, the levels of viral RNA did not differ among bronchial epithelial cells derived from healthy subjects, asthmatics, and patients with COPD (16). Sykes et al. reported that virus release following infection with RV 16 was not significantly different between subjects with and without asthma at any time point, showing the highest RV RNA levels at 48 h post-infection (23). Another study on RV 16 reported that RV 16 infection rates did not differ between allergic and non-allergic epithelial cells (24). A recent study showed no significant difference in the infection rate of RV 16 between epithelial cells derived from turbinate tissue of normal subjects and patients with CRSwNP (25). Another study showed that the replication of RV1B was not different in epithelial cells between patients with allergic rhinitis and healthy subjects (26). In contrast, in a mouse model of chronic allergic rhinosinusitis, the RV1B infected area was significantly larger than that in control mice (27). RV – A16 infected epithelial cells derived from patients with asthma had increased viral replication compared to that in healthy subjects (13). Taken together, these data indicate that the replication rates of respiratory viruses in sinonasal epithelial cells may depend on the type of virus and the cultured cells. Veerati et al. suggested that these discrepancies may be explained by disease characteristics such as disease severity, inflammatory phenotypes, and culture conditions (16). More comparable studies are required to address these issues.

Our study failed to find any evidence of deficient induction of IFN-β, -λ1, -λ2, and ISGs in cultured inflammatory epithelial cells in response to RV 16 infection and poly(I: C) treatment. Our time-course analysis demonstrated that RV 16 infection in cultured normal and inflammatory epithelial cells enhanced maximal induction of IFN-β, -λ1, -λ2, and ISGs mRNA and protein levels at 48 h post-infection. Interestingly, these findings were accompanied by viral replication rates which reached a peak 48 h after RV 16 exposure in both groups of epithelial cells. Furthermore, anti-viral interferon production after treatment with poly (I: C) also showed no significant difference between both groups of epithelial cells, although the maximal levels of anti-viral interferon release were found at 24 h after poly (I: C) treatment. These findings suggest that RV- or TLR3 agonist-induced anti-viral interferon release is not delayed or deficient in patients with CRSwNP compared with healthy controls. These data are in line with a previous *in vitro* study, that reported that cultured bronchial epithelial cells of asthmatic patients showed higher levels of IFN-β than cultured normal epithelial cells from healthy subjects (28). However, there was no significant difference in the degree of RV replication between patients with asthma and healthy donors (28). These findings are in line with those of other studies in which antiviral-interferon release following RV infection was unaltered in cells isolated from patients with respiratory disease (29–31). To date, there is limited data on anti-viral interferon release following RV infection in sinonasal epithelial cells derived from patients with CRSwNP. Contrary to our finding, Kim et al. reported that RV 16 infection in patients with CRS resulted in slightly impaired secretion of IFN-β compared with that in controls (25). A study using nasal epithelial cells of turbinate mucosa reported that the replication of RV1B was significantly increased in nasal epithelial cells of patients with allergic rhinitis and the mean IFN-λ1 mRNA expression was lower in RV1B-infected cells (26). However, poly (I: C)-treated nasal epithelial cells induced a similar IFN-λ1 generation in patients with allergic rhinitis and healthy subjects (26). Taken together with these results, although impaired anti-viral interferon responses to respiratory viral infection have been consistently observed in asthma and COPD (13–17), a similar disease process may not be frequently found in CRS. Further research is required to clarify these issues.

The activation of TLR3, RIG-I, and MDA5 is required for anti-viral interferon production in RNA virus-infected cells (7–9). Therefore, the next experiment was conducted to evaluate whether the expression levels of TLR3, RIG-I, and MDA5, and the activation of NF-κB and IRF3 are deficient or delayed in RV 16-infected or poly (I: C)-treated inflammatory epithelial cells which were derived from patients with CRSwNP. As expected, the expression levels of RIG-I, MDA5, TLR3, and the phosphorylated forms of NF-κB and IRF3 were not significantly different between both groups of epithelial cells at any time point after RV 16 infection or poly (I: C) treatment. Time course analysis showed that the levels of pattern recognition receptors and the phosphorylated forms of NF-κB and IRF3 peaked 48 h after RV 16 infection and 24 h after poly (I: C) treatment in both groups of epithelial cells and activated without delay in inflammatory epithelial cells. Taken together, these data indicate that the expression of TLR3, RIG-I, MDA5, and the phosphorylated forms of NF-κB and IRF3 are normally induced in inflammatory epithelial cells derived from patients with CRSwNP when infected with RV 16 infection or treated with poly (I: C). Therefore, it is possible that the anti-viral interferon response induced by the stimulation of TLR3, RIG-I, and MDA5 is not deficient in patients with CRSwNP because of the intact signaling pathways. Similar to our own findings, their expression was not different in patients with asthma compared to healthy subjects in both their airways and cultured bronchial epithelial cells from both subjects (32). RV infection enhances the robust expression of MDA5 and RIG-I in bronchial epithelial cells (32). The expression of RIG-I and MDA5 was not impaired despite the deficiency of RV-induced interferon in bronchoalveolar lavage cells from asthmatic patients (33). However, in these studies, RV- induction of these molecules did not appear to explain the deficient RV-induced interferon secretion in patients with respiratory disorders.

In summary, this is the first report to evaluate RV 16 replication rates, secretion of anti-viral interferons, expression levels of pattern recognition receptors, and key signaling molecules in normal and inflammatory sinonasal epithelial cells which were infected with RV 16 or treated with poly (I: C). We found no difference in the RV16 replication rates between both groups of epithelial cells. Time-course analysis showed that the expression of type I (IFN-β) and III IFN (IFN-λ1 and -λ2) and ISGs reached peak levels 48 h after RV 16 infection and 24 h after poly (I: C) treatment in both groups of epithelial cells. Similar patterns were observed in the expression levels of TLR3, RIG-I, and MDA5. NF-κB and IRF3 were activated without delay or deficiency in the inflammatory epithelial cells compared with normal epithelial cells. These results provide evidence that the anti-viral interferon response to RV 16 infection is neither deficient nor delayed in patients with CRSwNP. However, some limitations exist in this study. Although there were no proper animal models for CRSwNP, the antiviral interferon response to RV 16 needs to be confirmed in animal model as well as in clinic field.

## Data availability statement

The original contributions presented in the study are included in the article/**Supplementary Material**. Further inquiries can be directed to the corresponding author.

## Ethics statement

The studies involving human participants were reviewed and approved by The institutional review boards for human subjects at Korea University hospital approved the protocols and the informed consent form. The patients/participants provided their written informed consent to participate in this study.

## Author contributions

SaL conceived and designed the study and performed experiments. MH and TK performed the collection of samples and experiments and analyzed data. TL, DL, JP, SeL, and TK performed the collection of samples, the performance of experiments, analyzed data and contributed to data visualization. All authors wrote the manuscript and approved the final manuscript.

## Funding

This work was supported by The Basic Science Research Program through the National Research Foundation of Korea (2022R1A2C1003461).

## Acknowledgments

We would like to thank all participants for the sample provided.

## Conflict of interest

The authors declare that the research was conducted in the absence of any commercial or financial relationships that could be constructed as a potential conflict of interest.

## Publisher’s note

All claims expressed in this article are solely those of the authors and do not necessarily represent those of their affiliated organizations, or those of the publisher, the editors and the reviewers. Any product that may be evaluated in this article, or claim that may be made by its manufacturer, is not guaranteed or endorsed by the publisher.
