# Supplementary figures and images for: Rhinovirus-induced anti-viral interferon secretion is not deficient and not delayed in sinonasal epithelial cells of patients with chronic rhinosinusitis with nasal polyp
Source: Front Immunol. 2022 Oct 21;13:1025796. doi: 10.3389/fimmu.2022.1025796 (PMC9635927; doi:10.3389/fimmu.2022.1025796)

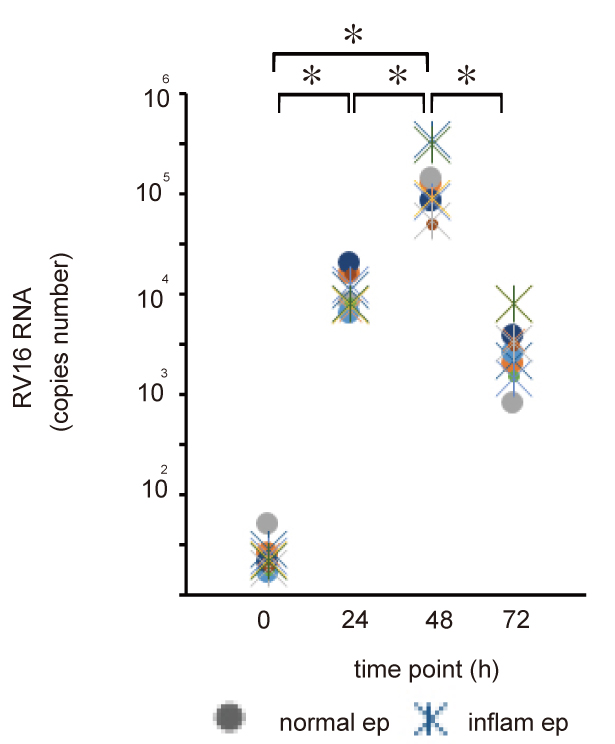

Supplement: Supplementary file 1 [file Image_1.tif]

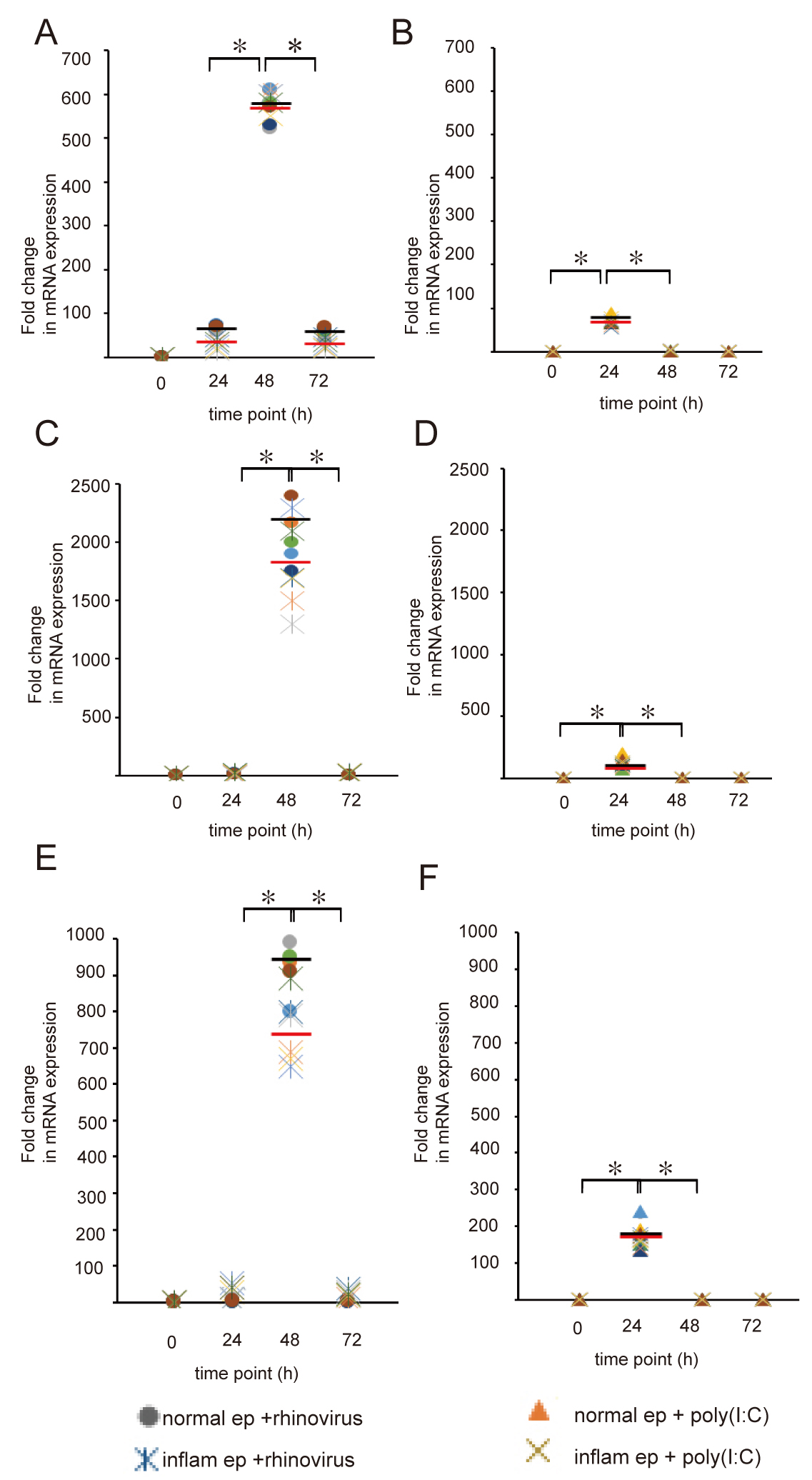

Supplement: Supplementary file 2 [file Image_2.tif]

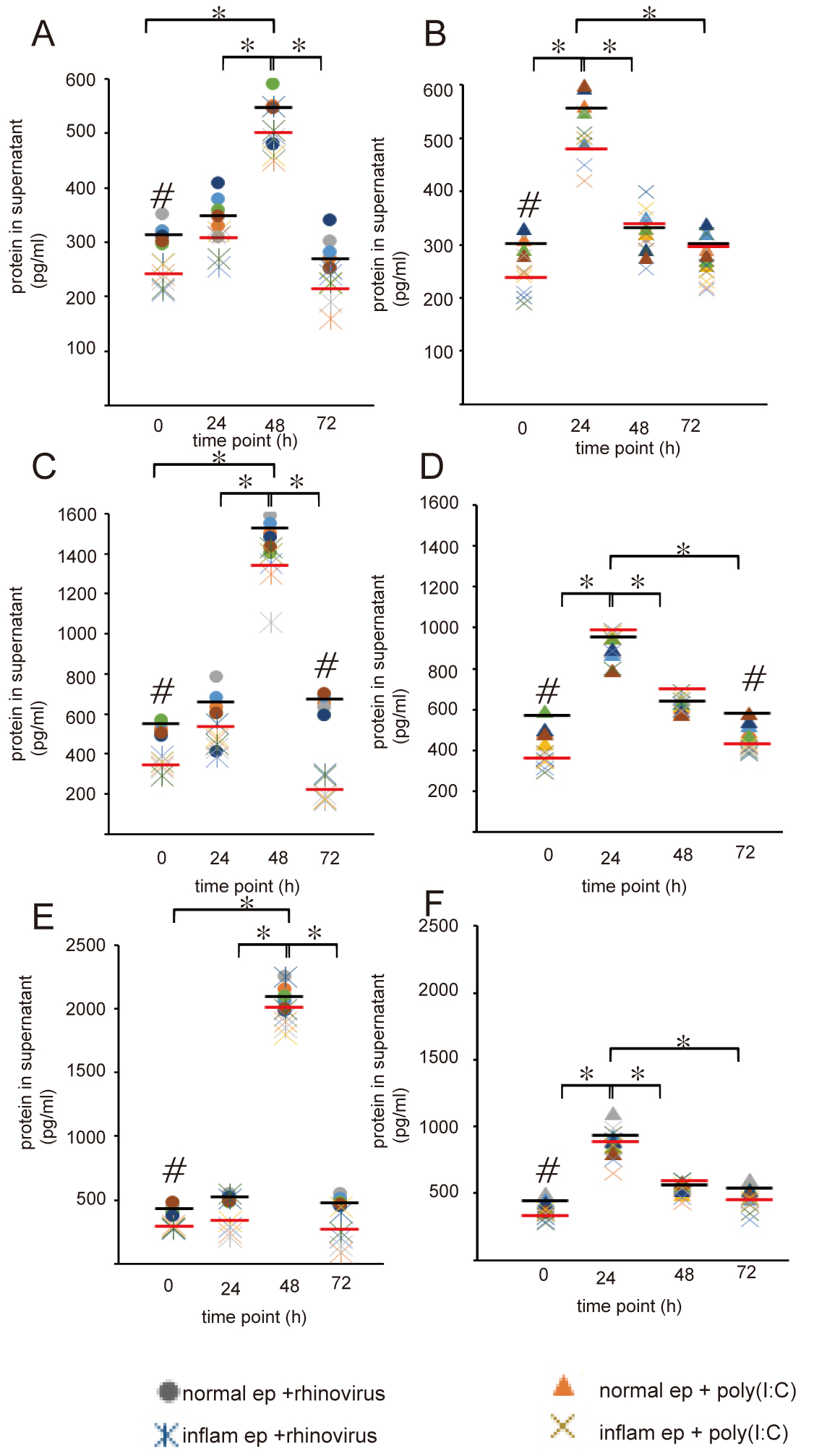

Supplement: Supplementary file 3 [file Image_3.tif]

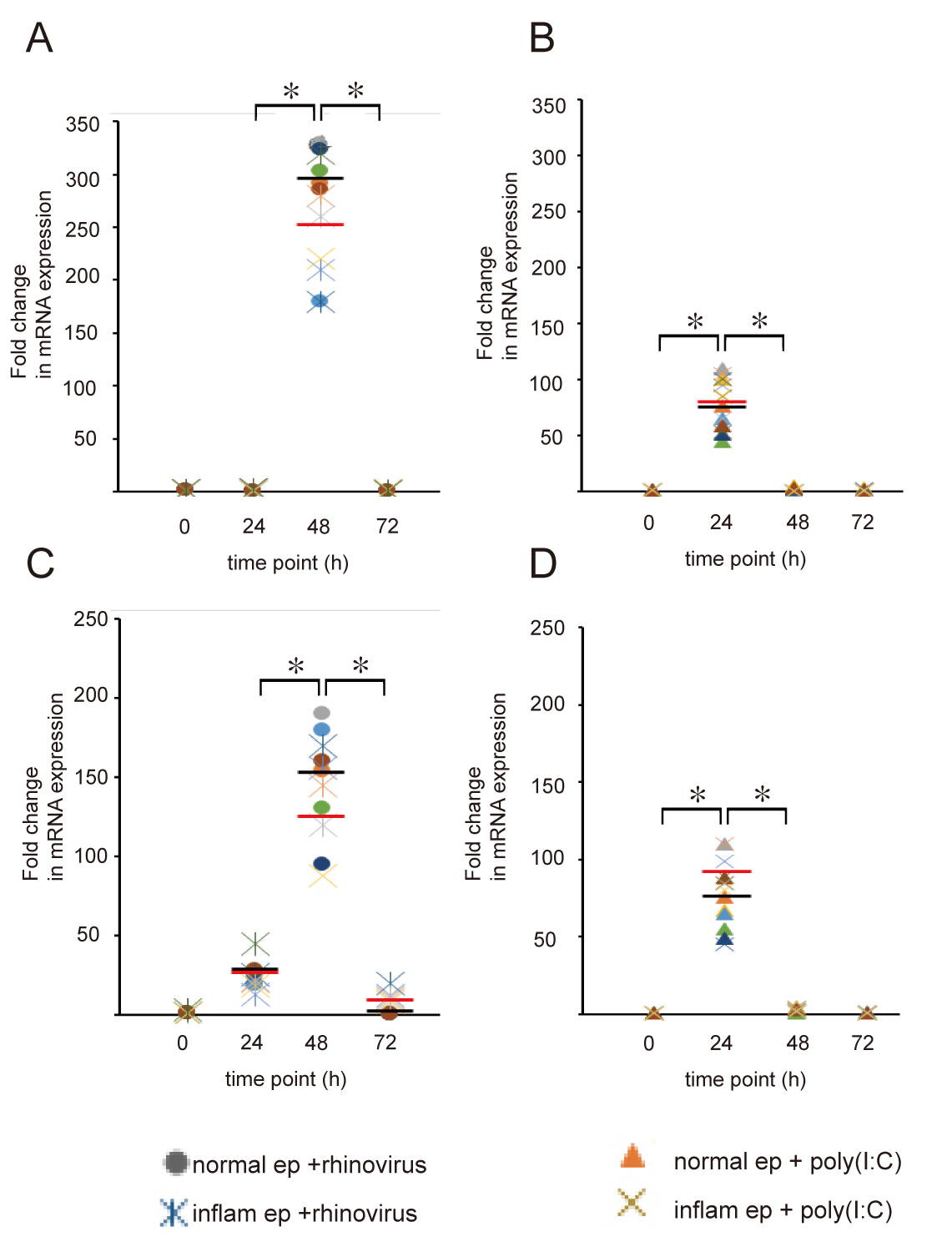

Supplement: Supplementary file 4 [file Image_4.tif]

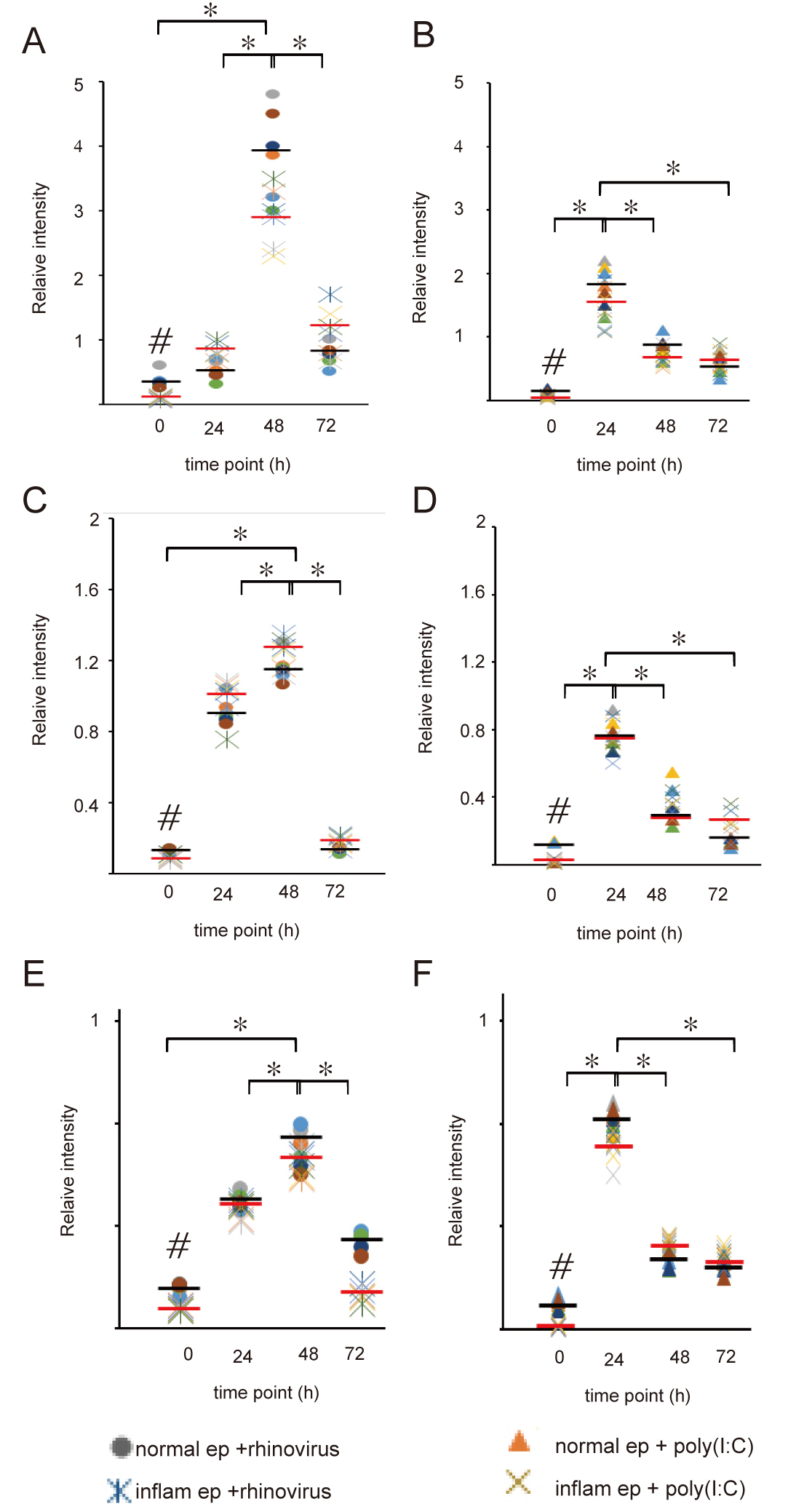

Supplement: Supplementary file 5 [file Image_5.tif]

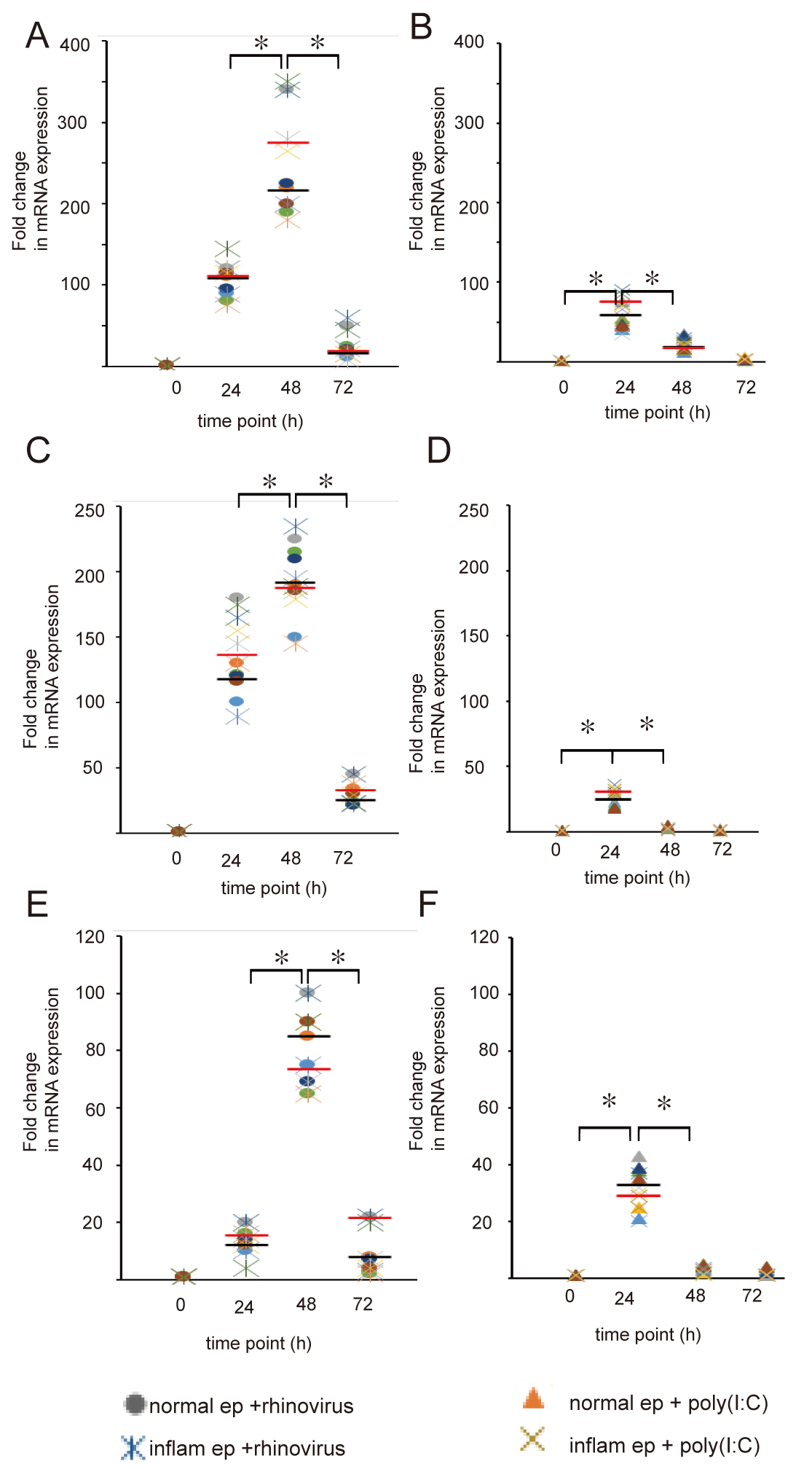

Supplement: Supplementary file 6 [file Image_6.tif]

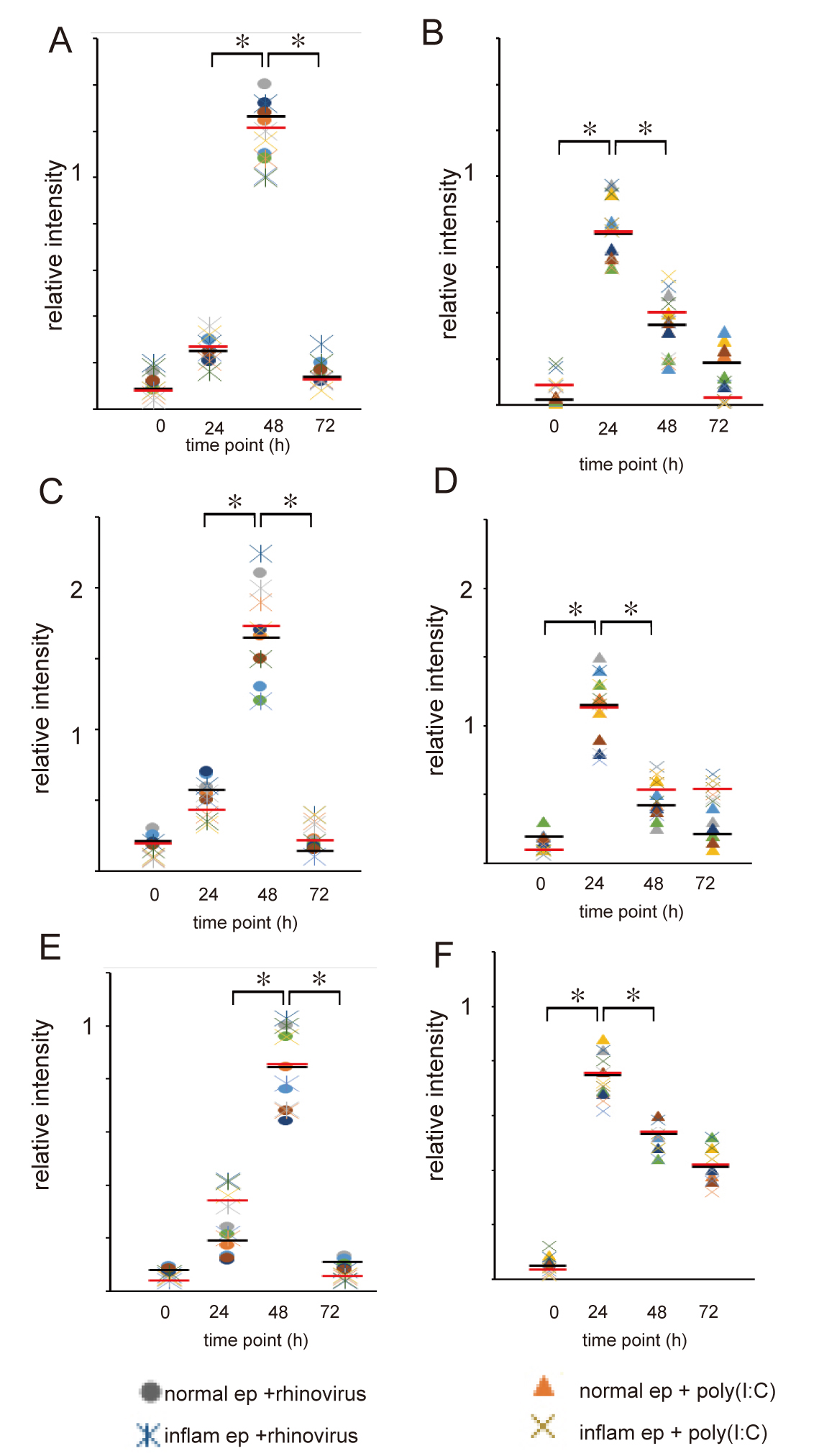

Supplement: Supplementary file 7 [file Image_7.tif]

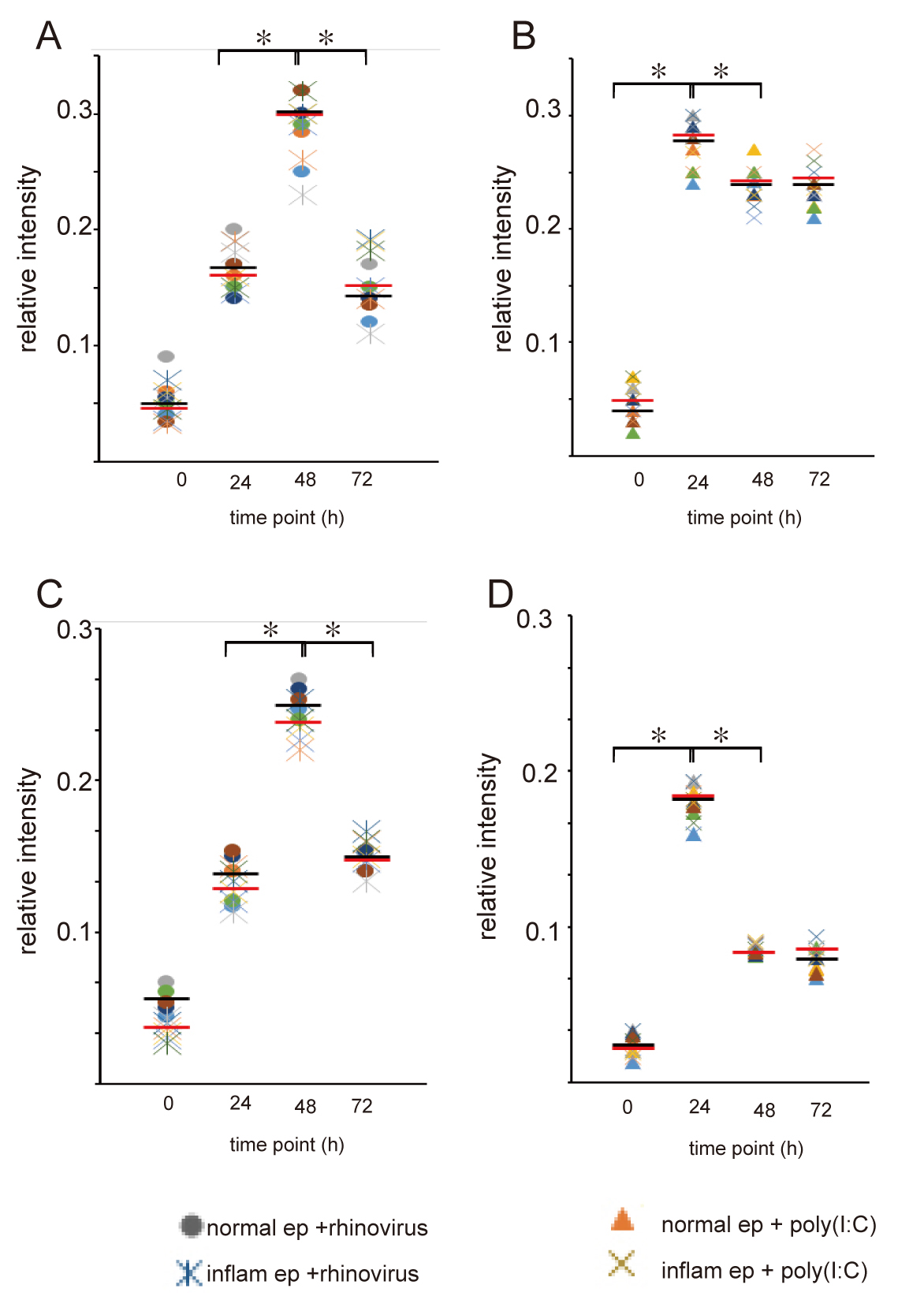

Supplement: Supplementary file 8 [file Image_8.tif]
