# Supplementary material for: Rhinovirus-induced anti-viral interferon secretion is not deficient and not delayed in sinonasal epithelial cells of patients with chronic rhinosinusitis with nasal polyp
Source: Front Immunol. 2022 Oct 21;13:1025796. doi: 10.3389/fimmu.2022.1025796 (PMC9635927; doi:10.3389/fimmu.2022.1025796)
Supplement: Supplementary file 9 [file Table_1.docx]

Supplementary table 1. Characteristics of the study group

Healthy control subjects CRSwNP

Septal deviation Blowout fracture

(n = 50) (n=15) (n = 59)

Sex

Male 45 12 49

Female 5 3 10

Mean age (Y) 43.33±5.02 29 ± 5.8 45.76 ± 14.84

SNOT-20

Score* 10.67 ± 3.48 8.14 ± 2.58 39.63± 7.0

Computed

tomographic

grade* 1.78 ± 0.38 1.89±0.25 14.5 ± 3.12

Endocopy

Score* 0 0 7.43±3.14

*SNOT-20 score, computed tomographic grades, and endoscopy score in patients with patients with CRSwNP are higher than control subjects (p<.05). SNOT-20 score; the average 20-item Sino-Nasal Outcome Test score.
