# Supplementary material for: Rhinovirus-induced anti-viral interferon secretion is not deficient and not delayed in sinonasal epithelial cells of patients with chronic rhinosinusitis with nasal polyp
Source: Front Immunol. 2022 Oct 21;13:1025796. doi: 10.3389/fimmu.2022.1025796 (PMC9635927; doi:10.3389/fimmu.2022.1025796)
Supplement: Supplementary file 10 [file Table_2.docx]

The relative gene expression levels of IFN-β, IFN-λ1, and IFN-λ2 in normal and inflammatory epithelial cells after RV 16 infection and poly (I:C) treatment, which was analyzed by real time PCR

0 h 24 h 48h 72 h

IFN-β

Normal ep + rhino 11.01±1.99 * 65.11±32.45 578.34±76.22 54.89±34.67

Inflam ep + rhino 1 30.34±34.56 564.33±56.21 27.88±45.22

Normal ep +poly(I:C) 12.11±1.23* 75.89±21.11 11.12±3.45 10.12±3.33

Inflam ep + poly(I:C) 1 66.23±24.67 9.53±3.22 9.91±0.34

IFN-λ1 (pg/ml)

Normal ep + rhino 13.11±3.66* 15.34±2.34 2164.45±345.22 22.33±1.24

Inflam ep + rhino 1 19.22±2.11 1746.21±754.67 23.66±15.11

Normal ep +poly(I:C) 14.22±2.45.* 129.46±23.45 1.28±0.07 1.11±1.10

Inflam ep + poly(I:C) 1 115.67±35.66 1.13±0.09 1.35±0.11

IFN-λ2 (pg/ml)

Normal ep + rhino 14.23±2.85* 34.53±2.22 934.22±121.23 22.34±1.23

Inflam ep + rhino 1 39.45±45.45 743.78±150.34 23.56±12.35

Normal ep +poly(I:C) 15.33±3.45* 188.64±64.33 1.43 ±1.22 1.23±0.08

Inflam ep + poly(I:C) 1 162.98±63.12 1.02±1.09 1.34±0.09

* indicates statistical difference (P<0.05) in the expression levels of IFN-β, IFN-λ1, and IFN-λ2 mRNA between normal and inflammatory epithelial cells at 0 h.
