# Supplementary material for: Rhinovirus-induced anti-viral interferon secretion is not deficient and not delayed in sinonasal epithelial cells of patients with chronic rhinosinusitis with nasal polyp
Source: Front Immunol. 2022 Oct 21;13:1025796. doi: 10.3389/fimmu.2022.1025796 (PMC9635927; doi:10.3389/fimmu.2022.1025796)
Supplement: Supplementary file 11 [file Table_3.docx]

The expression levels of IFN-β, IFN-λ1, and IFN-λ2 proteins in normal and inflammatory epithelial cells after RV 16 infection and poly (I:C) treatment which was analyzed by ELISA

0 h 24 h 48h 72 h

IFN-β (pg/ml)

Normal ep + rhino 322.25 ± 33.5 * 373.56 ± 54.22 553.12 ± 47.89 265.65 ±65.78

Inflam ep + rhino 250. 34 ± 23.38 290. 34 ± 73.38 491.34 ±75.89 213.56 ± 75.21

Normal ep +poly(I:C) 310.76 ± 29.45*  561.67± 54.21 320.22 ± 55.89 290.34 ±67.98

Inflam ep + poly(I:C) 235. 45 ± 35,89 490.22 ± 66.78 322.56 ± 78.45 294.23 ±56.11

IFN-λ1 (pg/ml)

Normal ep + rhino 574.10 ± 25.33* 632.11 ± 172.12 1524.26 ± 78.22 692.32 ± 64.77*

Inflam ep + rhino 352.33 ± 34.56 475.33 ± 173. 44 1300. 45 ±153.44 300.23 ± 33.12

Normal ep +poly(I:C) 490.22 ± 63.44.* 890.12 ± 102.45 632.45 ± 89.34 587.66±55.67*

Inflam ep + poly(I:C) 250. 45 ± 45,89 870.22 ± 66.78 602.56 ± 78.45 414.23 ±76.11

IFN-λ2 (pg/ml)

Normal ep + rhino 432.35 ±65.11* 521.23±33.45 2154.2±242.11 489.43±55.11

Inflam ep + rhino 291.11±12.32 453.22±163.22 1956.33±232.33 354.33±102.33

Normal ep +poly(I:C) 403.55±33.45* 911.23±398.56 554.33±65.22 512.76±75.22

Inflam ep + poly(I:C) 342.55±12.11 856.76±266.78 554.56±67.43 413.33±89.22

* indicates statistical difference (P<0.05) in the expression levels of IFN-β, IFN-λ1, and IFN-λ2 mRNA between normal and inflammatory epithelial cells at 0 and 72 h
